# Challenges of Empirical Antibiotic Therapy for Community-Acquired Pneumonia in Children

**DOI:** 10.1016/j.curtheres.2017.01.002

**Published:** 2017-01-16

**Authors:** Charlene M.C. Rodrigues

**Affiliations:** 1 Department of Zoology, University of Oxford, Oxford, United Kingdom; 2 Department of Paediatric Immunology and Infectious Diseases, Newcastle upon Tyne Hospitals Foundation Trust, Great North Children’s Hospital, Newcastle upon Tyne, United Kingdom

**Keywords:** antibiotics, chest infection, community-acquired pneumonia, empirical, lower respiratory tract infection, management

## Abstract

**Background:**

Community-acquired pneumonia (CAP) is a leading cause of morbidity and mortality globally, responsible for more than 14% of deaths in children younger than 5 years of age. Due to difficulties with pathogen identification and diagnostics of CAP in children, targeted antimicrobial therapy is not possible, hence the widespread use of empirical antibiotics, in particular penicillins, cephalosporin, and macrolides.

**Objectives:**

This review aimed to address medical, societal, and political issues associated with the widespread use of empirical antibiotics for CAP in the United Kingdom, India, and Nigeria.

**Methods:**

A literature review was performed identifying the challenges pertaining to the use of widespread empirical antibiotics for CAP in children. A qualitative analysis of included studies identified relevant themes. Empirical guidance was based on guidelines from the World Health Organization, British Thoracic Society, and Infectious Diseases Society of America, used in both industrialized and resource-poor settings.

**Results:**

In the United Kingdom there was poor adherence to antibiotics guidelines. There was developing antibiotic resistance to penicillins and macrolides in both developing and industrialized regions. There were difficulties accessing the care and treatment when needed in Nigeria. Prevention strategies with vaccination against *Streptococcus pneumonia, Haemophilus influenza,* and measles are particularly important in these regions.

**Conclusions:**

Effective and timely treatment is required for CAP and empirical antibiotics are evidence-based and appropriate in most settings. However, better diagnostics and education to target treatment may help to prevent antibiotic resistance. Ensuring the secure financing of clean food and water, sanitation, and public health infrastructure are also required to reduce the burden of disease in children in developing countries.

## Introduction

In 2016, community-acquired pneumonia (CAP) remained an important cause of morbidity and mortality in both industrialized and developing countries.^1^ Between 2000 and 2010, pneumonia caused 14.1% (n = 1,071,000) of all deaths worldwide in children aged 1 month to 5 years, making it the single most significant disease.^2^ There are many factors that influence CAP incidence and disproportionately affect children in developing countries, including access to health care, vaccine implementation, living conditions, and nutrition (Table I). However, CAP remains a globally problematic disease and the barriers to overcoming its influences are multifactorial and varied across different regions of the world.

**Table I:** Estimated incidence of community-acquired pneumonia in industrialized and developing regions of the world. Estimated incidence of community-acquired pneumonia in industrialised and developing regions of the world, reported by the World Health Organisation.^1^

Region	Incidence (episodes per child-year)	Number of new cases per year (millions)
Southeast Asia	0.36	60.95
Africa	0.33	35.13
East Mediterranean	0.28	19.67
Western Pacific	0.22	29.07
Americas	0.10	7.84
Europe	0.06	3.03

### Why do we need empirical antibiotics for CAP?

The use of empirical antibiotics is inevitable due to the challenges of accurately diagnosing CAP and identifying the causative organism. Current guidelines for the management of CAP in children have been produced by the World Health Organization (WHO),^3^ British Thoracic Society (BTS),^4^ and Infectious Diseases Society of America^5^ (this discussion will not include the treatment of neonates, immunocompromised patients, or those with underlying respiratory conditions). These guidelines have been written by clinicians and academics in the fields of respiratory medicine, infectious diseases, microbiology, and epidemiology, with substantial review of the literature. Further Cochrane systematic reviews have also extensively reviewed the body of evidence to optimize empirical guidance.[6], [7], [8], [9] They recognize the literature in both industrialized and developing countries is lacking and in need of good epidemiologic data and large, multicenter, randomized controlled trials (RCTs).

The consensus recommendations from these guidelines suggest first-line antibiotics (eg, amoxicillin and cephalosporins) for CAP and severe CAP based on the most frequently identified bacteria (ie, *Streptococcus pneumonia*) and the use of oral antibiotics in preference to intravenous (IV) unless there is severe pneumonia or the child is unable to tolerate oral antibiotics, is vomiting, or has complications.^3^ Therefore, the severity of CAP must be assessed to decide whether or not the child needs treatment and if so the most suitable mode of antibiotic administration.

The main aim of antimicrobial agents is to limit progression to severe or very severe CAP and the associated mortality. However, given the ongoing contribution of CAP to global morbidity and mortality, despite global implementation of empirical management strategies, this review aims to analyze the medical, societal, and political challenges facing the widespread use of such guidelines. Region-specific issues with empirical management were evaluated with respect to 3 countries: the United Kingdom representing industrialized regions and India and Nigeria representing the 2 countries with highest estimated incidence of CAP in Asia and Africa, respectively.^2^

## Methods

A literature search was performed to address the hypothesis that the challenges with widespread empirical antibiotic use for children with CAP are diverse in the United Kingdom, India, and Nigeria. Literature searches were done using PubMed and Scopus (April 2016) and only included studies published in English (there were no non-English studies identified in the searches). Search terms used included *UK* AND *Children* AND *Community-acquired pneumonia* AND *Antibiotics* (24 results); *India* AND *Children* AND *Community-acquired pneumonia* AND *Antibiotics* (23 results); *Nigeria* AND *Children* AND *Community-acquired pneumonia* AND *Antibiotics* (2 results), *United Kingdom* AND *Pneumonia* AND Children AND *Treatment* (391 studies), *India* AND *Pneumonia* AND *Children* AND *Treatment* (369 studies), and *Nigeria* AND *Pneumonia* AND *Children* AND *Treatment* (77 studies). The resulting 886 studies were screened, by title and abstract, for relevance using the following inclusion criteria: CAP national guidelines; antibiotic efficacy; mode of antibiotic administration; and implementation of CAP guidelines or medical, societal, financial, or cultural consequences of using empirical treatment for CAP in children. Exclusion criteria included studies of CAP in adults, complicated pneumonia; CAP occurring in regions outside of the United Kingdom, India, or Nigeria; and studies not relating to pneumonia. All included studies underwent a qualitative analysis of the complete article and were categorized into the following themes: antibiotic use and efficacy; mode of antibiotic administration; implementation of CAP guidelines; antibiotic resistance; and medical, societal, financial, and cultural influence of empirical CAP management. These themes are discussed according to the 3 countries below.

## Results and Discussion

### United Kingdom: Vaccination against bacterial pathogens and epidemiology

In the United Kingdom, 7-valent pneumococcal conjugate vaccine (PCV 7) was introduced into the national immunization schedule in September 2006 and replaced by PCV13 in April 2010. During 2012-2013, vaccine coverage in England reached 94.4% for primary immunization course PCV and 92.7% for the booster combined with *Haemophilus influenzae* type b (Hib)/meningococcal C.^10^ To identify the common pathogens responsible for CAP, a study of 160 children with clinically or radiologically confirmed CAP were investigated using a combination of blood culture, serology, and molecular methods for bacterial and viral isolation (Table II).^11^ The BTS guidance was published in 2011 (predated by guidance from 2002) and proposed amoxicillin as the first-line oral antibiotic, which has good efficacy against the most prevalent bacterial pathogens *S pneumoniae* and *H influenzae.*^12^ Amoxicillin is also well absorbed from the gut and its side effects are well tolerated.

**Table II:** Distribution of pathogens most frequently identified from studies within the geographic regions of the United Kingdom, India, and Nigeria.* This is not an exhaustive list of microbial population epidemiology. Adapted from references [11], [27], [34].

	United Kingdom	India	Nigeria
Viral aetiology			
Respiratory syncytial virus	21.2	24.1	30.4
Rhinovirus	8.5	10.5	
Human metapneumovirus	0.7	2.8	
Influenza A and B	7.4	3.5	17.3 (only A)
Bocavirus	3.3		
Adenovirus	6.9	3.7	
Parainfluenza	4.3 (types 1-4)	7.5	19.5 (type 3)
Bacterial aetiology			
*Streptococcus pneumoniae*	17.4	20.4	5.1
*Haemophilus influenzae*	2.3	8.2	
Group A *Streptococcus*	10.5		
*Staphylococcus aureus*	2.3	30.6	37.3
*Mycoplasma pneumoniae*	9.9	4.3 (serology)	
*Moraxella catharrhalis*	2.3		
*Klebsiella pneumoniae*	0.8	12.2	15.3

⁎ Values are presented as %.

### United Kingdom: Poor adherence to national guidelines

To evaluate implementation, a national audit from 2009-2012 reviewed the management of children older than age 1 year hospitalized with CAP and identified poor adherence to the new BTS guidance. Considering oral antibiotics, there was overuse of macrolides (35.2% of all oral prescriptions) and co-amoxiclav (34.2%) compared with amoxicillin (24.2%) in 2011-2012. The use of IV antibiotics included the most frequent use of co-amoxiclav (39.6%), cefuroxime (17.8%), amoxicillin (7.6%), and cefotaxime (6.3%).^13^ It was acknowledged that avoidance of amoxicillin could be due to previous primary care treatment before presentation to hospital and mode of administration was not collected for the first 2 years of the study. However, in view of the nonadherence surrounding IV antibiotics, further studies are required to reassure pediatric practitioners of the equivalence to oral regimens in severe CAP.

The PIVOT trial sought to add to the body of evidence as a nonblinded RCT of equivalence of oral and IV antibiotic therapy for hospitalized children with severe CAP. Children with clinically and radiologically confirmed CAP (n = 264) were randomized to 7 days of oral amoxicillin or IV benzylpenicillin (changing to oral amoxicillin but completing a total of 7 days’ therapy). The primary outcome measure of temperature <38°C was equivalent at 1.3 days (P = 0.03), with significantly longer hospital admissions with IV therapy (2.1 days vs 1.77 days; *P* < 0.001) and longer time on oxygen (20.5 vs 11.0 hours; *P* = 0.04).^14^

### United Kingdom: Cost implications of nonadherence to national guidance

The increased use of IV antibiotics also raises significant cost implications based on direct (ie, investigations, drugs, hospital admission, and staffing) and indirect (ie, parental time off work, travel, and parking) costs. Lorgelly et al^15^ performed a cost-minimization analysis alongside the PIVOT equivalence RCT and found that oral amoxicillin was more cost-effective than IV therapy for all except the sickest children. By reducing hospital stay and drug costs, there could be an overall saving between £473 and £518 per child as well as reducing the effects on society.^15^

### United Kingdom: Lack of evidence base for macrolides in Mycoplasma pneumoniae CAP

For older children, macrolides are considered first-line treatment if *Mycoplasma* or *Chlamydia* CAP is suspected.[4], [5] A US study following a well-established PCV and Hib vaccination program identified *M pneumoniae* as the most frequent bacterial cause in all age groups with radiologically confirmed CAP (except those aged younger than 2 years).^16^ There is currently a paucity of data from the United Kingdom to make informed decisions about the use of macrolides in all age groups. A Cochrane systematic review of treatment of *M pneumoniae* CAP found a lack of RCTs, difficulty in identifying *M pneumoniae* early in the disease course, poor sensitivity and specificity of current serologic testing, and analyses done on often small subgroups of patients.^9^ The Cochrane review concluded that there was limited evidence for optimizing antibiotic choices and focused on 1 study of azithromycin treatment (3 days a week, for 3 weeks) versus placebo for children with acute respiratory infections on a background of recurrent respiratory infections.^17^ Short-term clinical success (defined as resolution of presenting symptoms and no new symptoms) was more frequent in those treated with azithromycin and significant in those with an identified atypical organism. Long-term clinical success was significantly more frequent in the treatment arm, whether or not an organism was identified.^17^ These results highlight many research issues including; the challenges of M pneumoniae identification, M pneumoniae acting as a colonizer rather than a pathogen, or macrolides acting via another mechanism (eg, anti-inflammatory).^18^ Of further concern was the rise of macrolide-resistant *M pneumoniae*. By 2013, the rates of resistance were highest in Asia (estimates of up to 90% in Japan and 97% in China),^19^ but reports of macrolide-resistant *M pneumoniae* in Scotland identified 6 out of 32 samples from high-clinical-risk patients showing genotypic resistance (19%).^20^

### India: Vaccination against bacterial pathogens and epidemiology

The Indian Academy of Pediatrics recommended introduction of PCV10 and PCV13 into their national immunization program in 2013.^21^ However, their implementation has not yet begun,^22^ possibly highlighting the disconnect between health research, policy, and government funding. India is 1 of 75 countries receiving Global Alliance for Vaccine and Immunizations assistance for implementation of PCV into the national immunization schedule. According to surveillance data, PCV13 and PCV10 would cover 62.4% to 74.6% and 55.6% to 64.0% of *S pneumoniae* serotypes, respectively, based on invasive pneumococcal diseases serotype distribution.[23], [24] In December 2011, 2 states in India, Kerala and Tamil Nadu,^25^ introduced Hib vaccination into their universal immunization programs. Good safety profiles and efficacy add supporting evidence for the government to fund the vaccine throughout India.^21^ Obtaining estimates of bacterial CAP incidence in a country the size of India is a significant challenge in the absence of a public health body. In addition, there is a lack of molecular diagnostics for accurate etiologic studies, a situation acknowledged by the Global Approach to Biological Research, Infectious diseases and Epidemics in Low-income countries (GABRIEL) Network, whose pneumonia etiology data for 10 low-income countries (including India) are awaited.^26^ Results from a prospective etiology study from North India were published in 2015 (Table II).^27^

Barriers to optimal management in India are different, but not unique to resource-poor settings. These include delayed recognition of illness, severe disease at presentation to a medical practitioner, poor living conditions, malnutrition, availability of over-the-counter antibiotics, and antimicrobial resistance.^28^

### India: Antibiotic resistance to empirical antibiotics

WHO guidance is generally followed in India; hence, amoxicillin is the recommended first-line oral agent, with ampicillin and gentamicin for IV use where a child has severe CAP. However, before 2013, co-trimoxazole was the recommended first-line empirical oral antibiotic.^3^ In 2010-2011 a study in Bangalore identified nasopharyngeal carriage isolates in 190 children with 41.5% resistant to co-trimoxazole and 16.9% resistant to penicillin.^29^ Carriage isolates are used as a surrogate marker of disease isolates in this situation.^30^ When invasive pneumococcal diseases isolates (n = 40) were considered in the same population, resistance rates were higher: 77.5% to co-trimoxazole, 35% to penicillin, and 12.5% multidrug resistant to penicillin, co-trimoxazole, and ceftriaxone.^31^ Penicillin resistance is an evolving problem in India and it highlights the issues with using empirical WHO-guided regimens (previously co-trimoxazole, but now amoxicillin) at a time where circulating pneumococci in this region are becoming increasingly resistant.

### India: Factors relating to suboptimal social and health care infrastructure

Considering other risk factors, a small case-control study in Nagpur region identified infancy, no measles immunization by 9 months, severe malnutrition, severe tachypnea at presentation, hypoxemia at baseline, and bacteremia as factors predicting treatment failure in severe or very-severe CAP.^32^ The poor provision of clean water, sustenance, shelter, and sanitation are the focus of the United Nations Sustainable Development goals, but vaccination and public health infrastructure on a universal scale are dependent on political and health care sectors working in partnership.

### Nigeria: Vaccination against bacterial pathogens and epidemiology

The Hib and pneumococcal vaccines were introduced in 2012 and 2013, respectively.^33^ Despite this, the burden of CAP remains sizable, accounting for 16.4% of disease.^34^ From a study in an urban setting of 323 children with bronchopneumonia (72.4%), lobar pneumonia (20.4%), or both (7.1%), blood culture yield was high at 28.5%, despite 35.6% previous antibiotic use (Table II). Exposure to wood smoke, malnutrition, and bacteremia were risk factors associated with mortality in this cohort.^35^

### Nigeria: Societal and cultural practices lead to inequity in provision of antibiotic agents

Although Nigeria follows WHO pneumonia guidelines,^3^ availability, accessibility, and provision of WHO-recommended antibiotics to all children is not equitable. Maternal and child health interventions were part of the Millennium Development Goal 4 to optimize overall health. One particular measure included antibiotic administration for suspected pneumonia in children younger than age 5 years, with the aim of administering antibiotics in 90% of cases. The average coverage rate in Sokoko state region of northern Nigeria increased from only 13.5% to 26.06% between 2012 and 2013.^36^ Reasons for this include poor health infrastructure in health facilities and community programs, financial constraints, inefficiency of existing programs, as well as society-specific perceptions and cultures.

Societal factors and cultural practices in developing countries influence the use of antimicrobial agents and their efficacy. Examples of these include traditional healers and remedies, community health care workers, pharmacists, and drug vendors. WHO and United Nations Children’s Fund-supported Integrated Community Case Management packages were designed for pneumonia, diarrhea, and malaria to deliver health care away from health care facilities, improving access to medical interventions. Patent and proprietary medicine vendors (PPMVs) form part of the Integrated Community Case Management instigated by the Nigerian Ministry of Health to help deliver health care. However, these PPMVs are for-profit organizations that are highly accessible and a regular point of contact in rural or poor areas, which is a clear conflict of interest for communities that believe they have no other option for seeking health care. Evidence suggests that PPMVs’ knowledge of pneumonia was extremely poor and did not improve with formal pharmacy training (although this is not obligatory for practice). In addition, their activity included the illegal practice of selling antibiotics.^37^ Given the lack of understanding of pneumonia, it is difficult to ascertain if antibiotics are sold for children without CAP or withheld in cases of bacterial CAP, both having important consequences.

### Nigeria: Suboptimal parental engagement in CAP management

Parental involvement is crucial in the management of childhood illness. Nigerian parents have reported that even when they seek medical help at health facilities, supplies of antibiotic agents have run out and they must purchase them from PPMVs or elsewhere. This reduces trust in the medical facilities and influences future health-seeking behavior. When amoxicillin is prescribed and dispensed for home treatment, parents are responsible for administration at the appropriate dose and frequency. Evidence from Niger, and presumably an issue globally, suggests parents struggled to remember the instructions for use (in the absence of written instructions or illiteracy) and will discontinue antibiotics before completion of the full course.^38^

## Conclusions

The examples presented highlight the many difficulties faced when attempting to provide optimal management of CAP in children, especially those in resource-poor countries. These problems are not unique to the countries discussed here, but public health organizations must be mindful that antimicrobial therapy may not be reaching the children in need and if it does, it has questionable efficacy due to delayed diagnoses, incorrect administration, or antibiotic resistance. In order to improve the management of pediatric CAP, we need further research into clinical parameters that can accurately stratify CAP severity and more sensitive and specific diagnostics for identification of causative agents. However, this is only part of the solution, because optimizing implementation strategies, health education, and drug availability are paramount in high- and low-income countries alike. This must to be done on a global scale with an emphasis on improving the vaccination (pneumococcal, Hib, and measles) status and living conditions of the world’s poorest children. Only then will inroads be made into the burden of CAP in children.

## Conflicts of Interest

CMCR declares no conflict of interest. CMCR was responsible for the study design, literature review, data collection, data interpretation, and writing.
